# Microbial Metabolic Networks at the Mucus Layer Lead to Diet-Independent Butyrate and Vitamin B_12_ Production by Intestinal Symbionts

**DOI:** 10.1128/mBio.00770-17

**Published:** 2017-09-19

**Authors:** Clara Belzer, Loo Wee Chia, Steven Aalvink, Bhawani Chamlagain, Vieno Piironen, Jan Knol, Willem M. de Vos

**Affiliations:** a Laboratory of Microbiology, Wageningen University and Research, Wageningen, The Netherlands; b Department of Food and Environmental Sciences, University of Helsinki, Helsinki, Finland; c Nutricia Research, Utrecht, The Netherlands; d RPU Immunobiology, Faculty of Medicine, University of Helsinki, Helsinki, Finland; Max Planck Institute for Marine Microbiology

**Keywords:** *Akkermansia muciniphila*, anaerobes, butyrate, cross-feeding, intestine, microbiome, mucus, syntrophy

## Abstract

*Akkermansia muciniphila* has evolved to specialize in the degradation and utilization of host mucus, which it may use as the sole source of carbon and nitrogen. Mucus degradation and fermentation by *A. muciniphila* are known to result in the liberation of oligosaccharides and subsequent production of acetate, which becomes directly available to microorganisms in the vicinity of the intestinal mucosa. Coculturing experiments of *A*. *muciniphila* with non-mucus-degrading butyrate-producing bacteria *Anaerostipes caccae*, *Eubacterium hallii*, and *Faecalibacterium prausnitzii* resulted in syntrophic growth and production of butyrate. In addition, we demonstrate that the production of pseudovitamin B_12_ by *E. hallii* results in production of propionate by *A. muciniphila*, which suggests that this syntrophy is indeed bidirectional. These data are proof of concept for syntrophic and other symbiotic microbe-microbe interactions at the intestinal mucosal interface. The observed metabolic interactions between *A*. *muciniphila* and butyrogenic bacterial taxa support the existence of colonic vitamin and butyrate production pathways that are dependent on host glycan production and independent of dietary carbohydrates. We infer that the intestinal symbiont *A. muciniphila* can indirectly stimulate intestinal butyrate levels in the vicinity of the intestinal epithelial cells with potential health benefits to the host.

## IMPORTANCE

The intestinal microbiota is said to be a stable ecosystem where many networks between microorganisms are formed. Here we present a proof of principle study of microbial interaction at the intestinal mucus layer. We show that indigestible oligosaccharide chains within mucus become available for a broad range of intestinal microbes after degradation and liberation of sugars by the species *Akkermansia muciniphila*. This leads to the microbial synthesis of vitamin B_12_, 1,2-propanediol, propionate, and butyrate, which are beneficial to the microbial ecosystem and host epithelial cells.

## INTRODUCTION

The mammalian intestinal tract harbors complex microbial ecosystems that have been forged by millennia of coevolution between microbes and hosts. It is suggested that the evolution of metabolic interdependencies has led to strong deterministic processes that shape the composition of the microbiota during development (1). The diversity and richness of the gut microbiota within individuals, as well as the similarity in composition between individuals, are governed by several selective pressures within host habitats, such as diet (2, 3). Recent extreme interventions have illustrated the importance of dietary carbohydrates on the intestinal microbial community succession (4, 5). While dietary fibers affect substrate availability for the colonic microbiota, the mucus lining that covers the epithelial cells forms a consistent factor along its internal surface and is proposed to function as an endogenous prebiotic (6–9). The mucosal layer of the intestine is characterized by specific microbiota communities enriched with taxa affiliated with the family *Lachnospiraceae* (also known as *Clostridium* cluster XIVa) and the phylum *Verrucomicrobia* (10–15).

*Akkermansia muciniphila* is a mucus-colonizing member of the gut microbiota that has evolved to specialize in the degradation and utilization of host mucus, which it may use as the sole source of carbon and nitrogen (16, 17). Its mucin degradation activity leads to the production of 1,2-propanediol, propionate, and acetate (17). In addition, its mucosal foraging results in the availability of sugars liberated from mucus glycans and subsequent acetate production can stimulate coexistence of butyrogenic bacteria within the same mucosal niche (16). Microbe-produced short-chain fatty acids are described as major health-promoting compounds (18, 19). Because of its location close to the host cells, a symbiotic mucobiome could therefore be particularly important in fostering health in terms of nutrient exchange, communication with the host, regulation of the immune system, and resistance against invading pathogens.

Dietary intervention studies (13), *in vitro* mucosal model studies (20), and microbiota comparisons of gut lumen and epithelial biopsy specimens (11) have revealed strong cooccurrence of specific mucolytic bacteria (*A. muciniphila*, *Bacteroides* spp., and *Ruminococcus* spp.) and second-line butyrate producers (*Anaerostipes caccae*, *Eubacterium* spp., *Faecalibacterium prausnitzii*, and *Roseburia intestinalis*). This cooccurrence may be indicative of shared metabolic networks among the different microbial groups. *In vitro* isotope labeling has identified lactate and acetate as important precursors of butyrate production in human fecal samples (21). On top of this, kinetic modeling showed the likelihood for the dominant butyrate producers, such as *Anaerostipes coli* and *Eubacterium hallii*, to use short-chain fatty acids for butyrate production by utilizing lactate and acetate via the butyryl coenzyme A (CoA):acetate CoA transferase route, the main metabolic pathway for butyrate synthesis in the human colon (22).

In this study, we test the hypothesis that *A. muciniphila* can serve as the keystone species supporting a syntrophic network in a mucosal environment. Therefore, we studied the metabolic interactions between *A. muciniphila* and representative intestinal butyrate-producing bacteria; *F. prausnitzii* (representative of the family *Ruminococcaceae* also known as *Clostridium* cluster IV) and *A. caccae* and *E. hallii* (representatives of *Lachnospiraceae* also known as *Clostridium* cluster XIVa). The results indicate the existence of trophic chains on mucus between *A. muciniphila* and the butyrate-producing *F. prausnitzii* and *A. caccae*, while true bidirectional metabolic cross-feeding dependent on vitamin B_12_ was observed between *A. muciniphila* and *E. hallii*, indicative of a mutualistic symbiosis.

## RESULTS

### Growth and metabolism of intestinal butyrate producers on mucus or mucus-derived sugars.

In order to test whether *Akkermansia muciniphila* can serve as a keystone species in an environment where mucus is the main nutrient source, we first tested the ability of butyrate-producing mucosal colonizers to grow on mucus and mucus-derived sugars in the absence of *A. muciniphila*. When incubated in culture media with mucus as the sole carbon and nitrogen source, none of the butyrate-producing strains tested, *Anaerostipes caccae*, *Eubacterium hallii*, and *Faecalibacterium prausnitzii*, were able to grow or produce metabolites (see Table S2A in the supplemental material).

**FIG 1:**
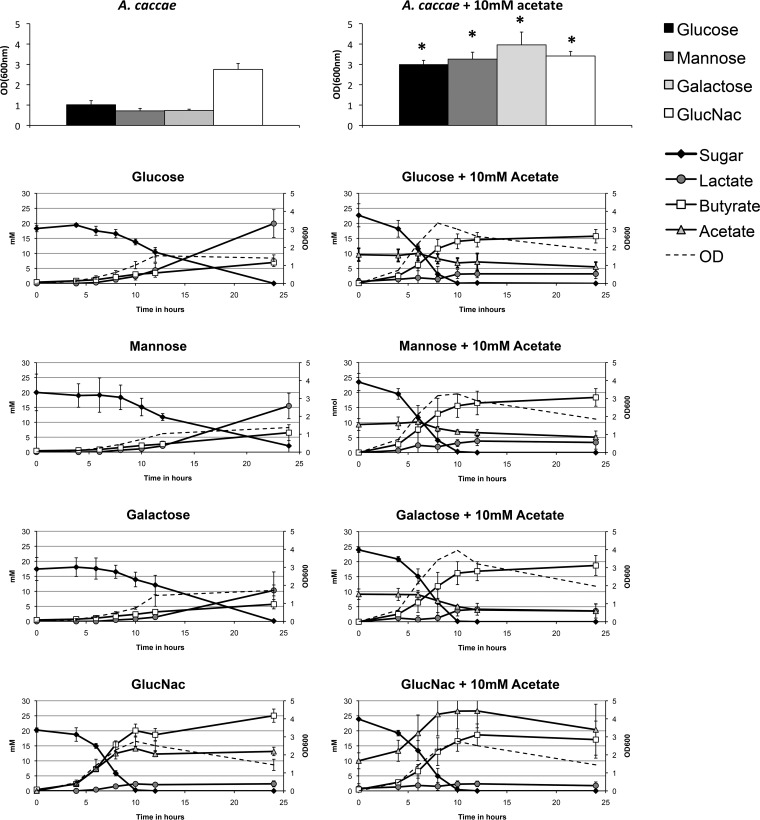
Metabolic activity of *A. caccae* on mucin-derived sugars. *A. caccae* was grown on monosaccharide present in the glycan chain of mucin. The OD_600_ values and HPLC profiles are shown for the sugars that resulted in positive growth. The sugars that gave positive test results were also used to perform experiments with the addition of 10 mM acetate. The graphs show the mean values for the experiments performed a minimum of three times in duplicate. Values that are significantly different (*P* < 0.05) in the presence of 10 mM acetate or absence of acetate are indicated by an asterisk. GlucNac, *N*-acetylglucosamine.

**FIG 2:**
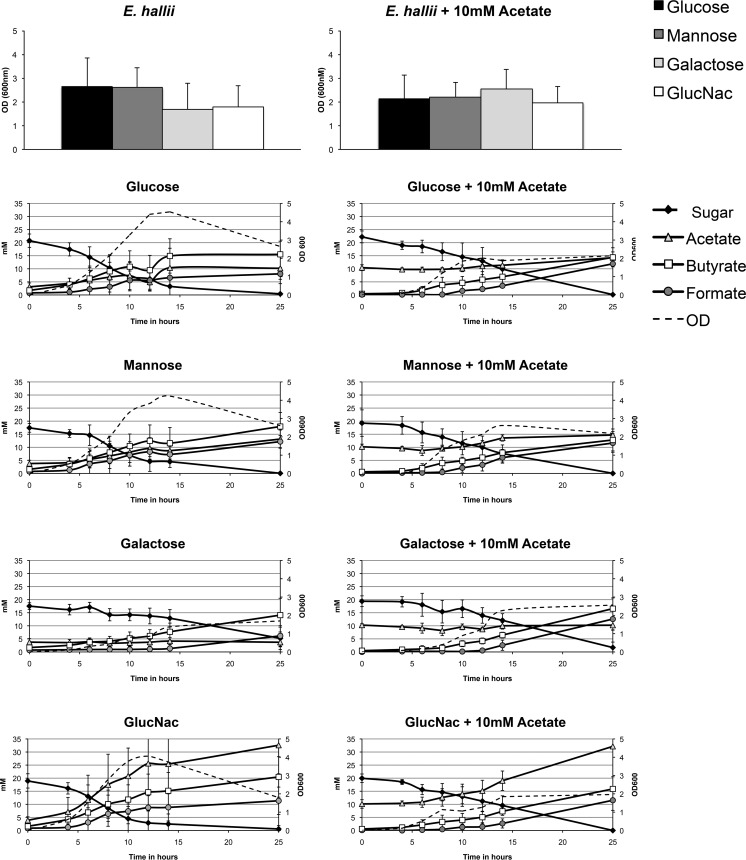
Metabolic activity of *E. hallii* on mucin-derived sugars. *E. hallii* was grown on monosaccharide present in the glycan chain of mucin. The OD_600_ value and HPLC profiles are shown for sugars that resulted in positive growth. The sugars that gave positive test results were also used to perform experiments with the addition of 10 mM acetate. The graphs show the mean values for the experiments performed a minimum of three times in duplicate.

The mucin sugars d-galactose, d-mannose, GlcNAc, GalNAc, and l-fucose and the non-mucin sugar glucose were subsequently tested as possible carbon sources for each butyrate-producing species. Minimal media used for the bacteria differed as a result of different minimal requirements for protein and spore elements (see Materials and Methods for details on the composition of the media). *F. prausnitzii* is known to be able to grow on GlcNAc and galactose (23). In addition, we tested the growth of *F. prausnitzii* on mannose and GalNAc, but no growth was observed (Table S2B). *A. caccae* was observed to use glucose, d-mannose, d-galactose, and GlcNAc for growth, and the main fermentation products were acetate, butyrate, and lactate (Fig. 1). The highest *A. caccae* cell numbers and acetate production were reached with GlcNAc, possibly due to the fact that fermentation of this amino sugar can replace the need for acetate in the medium (Fig. 1). *E. hallii* showed the same preference for sugars as *A. caccae* did, resulting in growth on glucose, d-mannose, d-galactose, and GlcNAc (Fig. 2). The main fermentation products of *E. hallii* were observed to be acetate, butyrate, and formate. Again GlcNAc resulted in the highest production of acetate and butyrate compared to the other sugars, but this was not accompanied with increased cell numbers of *E*. *hallii* (Fig. 2).

### Acetate enhances growth of *A. caccae* but not *E. hallii* on mucin-derived sugars.

The average production of 10 mM acetate by *A. muciniphila* grown in medium containing mucin could serve as the substrate for growth of butyrogens. Therefore, we added 10 mM acetate to cultures growing on glucose, d-mannose, d-galactose, and GlcNAc. In the case of *A. caccae*, this did indeed lead to the production of butyrate, acetate, lactate, and formate as measured in a minimal medium. Furthermore, these butyrate production levels were significantly higher than the observed butyrate production without added acetate (Fig. 1).

Weak growth of *A. caccae* on l-fucose was observed after the addition of acetate but without detected metabolite production. Acetate alone did not support growth (Table S2C). The addition of acetate to the growth media of *E. hallii* did not result in differences in growth or metabolite profile, possibly due to its own production of acetate (Fig. 2).

The overall fermentation efficiency was determined by calculating the carbon balance at each monosaccharide condition. The recovery of carbon atoms varied in between 70 and 100%, depending on the biomass produced that explains the loss (Tables 1 and 2).

**TABLE 1:** Carbon balance of *A. caccae* on mucin-derived sugars with or without acetate

Sugar	No. of carbons (mM)							Carbon recovery (%)	
	Substrates		Products						
	Sugar	Acetate	Lactate	Acetate	Butyrate	Formate	CO_2_	Avg	SD
Glucose	110		60		26		24	101	13
Glucose + 10 mM acetate	136	8	8		62	2	82	71	0
					
Mannose	121		55		27		27	85	12
Mannose + 10 mM acetate	140	8	10		73	2	76	78	8

Galactose	99		38		26		22	88	10
Galactose + 10 mM acetate	144	11	11		75	2	59	77	12

GlcNAc	162		7	26	98		27	98	2
GlcNAc + 10 mM acetate	192		5	31	84	3	34	81	11

**TABLE 2:** Carbon balance of *E. hallii* on mucin-derived sugars with or without acetate

Sugar	No. of carbons (mM)							Carbon recovery (%)	
	Substrates		Products						
	Sugar	Acetate	Lactate	Acetate	Butyrate	Formate	CO_2_	Avg	SD
Glucose	122			14	55	7	27	87	30
Glucose + 10 mM acetate	133			8	56	12	29	79	18

Mannose	106			19	66	12	24	117	24
Mannose + 10 mM acetate	115			9	49	12	26	85	21

Galactose	74	0,1			50	5	16	96	29
Galactose + 10 mM acetate	106	0,1			64	13	24	93	14

GlcNAc	147			57	76	11	25	116	40
GlcNAc + 10 mM acetate	160			44	61	11	27	90	19

### Mucus-induced trophic chains of *A*. *muciniphila* and butyrate producers *A*. *caccae*, *E. hallii*, and *F. prausnitzii* results in butyrate production

**FIG 3:**
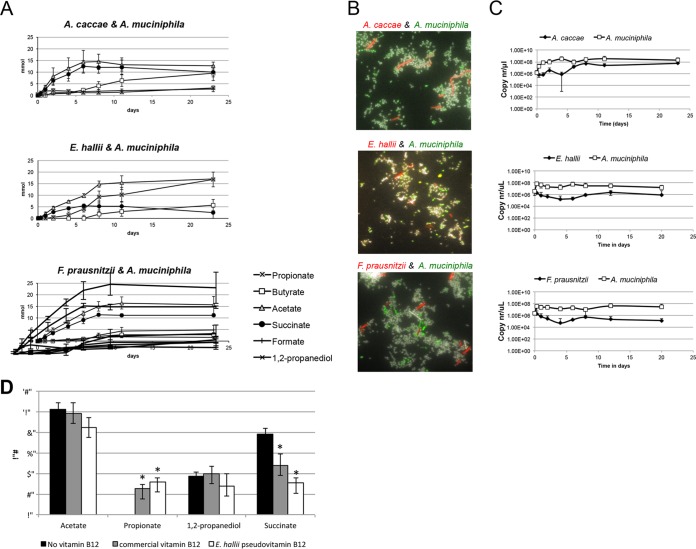
*A. muciniphila* degradation and fermentation of mucus enables cross-feeding by the butyrate-producing gut isolates. (A to C) Cocultures of *A. muciniphila* with butyrate-producing isolates were performed and measurements of product formation and consumption (A), FISH staining (B), and Q-PCR (C) were performed. (D) Measurement of *A. muciniphila* metabolites on mucus-containing media without the addition of vitamin B_12 _or with vitamin B_12_ from *E. hallii* or pseudovitamin B_12_ from *E. hallii*. The graph shows the mean values for the experiment performed a minimum of three times in duplicate. Asterisks indicate a significant difference (*P* < 0.05) compared to the condition without vitamin B_12_ added.

After the monoculture experiments, a series of cocultures of approximately equal amounts of *A. muciniphila* and butyrate producers were set up to test whether sugars and acetate produced as a result of mucin degradation by *A. muciniphila* would enable butyrate production of the chosen isolates. Remarkably, this coculturing on mucin-containing media supported growth and butyrate production for all three tested species (Fig. 3). *A. caccae* produced butyrate in levels comparable to those found in the monoculture conditions that were supplemented with acetate. Similarly, *F. prausnitzii* also produced butyrate in coculture with *A. muciniphila* and also produced 5 mM formate indicative of acetate consumption. Butyrate levels produced by *E*. *hallii* were in the range of what was seen in the monocultures growing on single sugars. The pH was monitored in all experiments and stayed around pH 6.5 throughout the experiments. Determination by quantitative PCR (Q-PCR) and qualitative presence (fluorescent *in situ* hybridization [FISH]) of the butyrate-producing species within the cocultures indicated a difference in abundance of the butyrate producers of several log units compared to the abundance of *A. muciniphila* (Fig. 3 and Table S1). The abundance of *A. caccae* increased 100-fold over the first 8 days of incubation based on the increase in its 16S rRNA gene copy number. Maximum butyrate levels were reached after 11 days of incubation. In contrast to the results for cultures, no lactate was measured during the cross-feeding experiments with *A. caccae*. Both Q-PCR and FISH results indicated a ratio of *A*. *muciniphila* to *A. caccae* of approximately 100:1.

10.1128/mBio.00770-17.1 TABLE S1  Qualitative measurements in coculture experiments by FISH. Download TABLE S1, PDF file, 0.04 MB. Copyright © 2017 Belzer et al. 2017 Belzer et al. This content is distributed under the terms of the Creative Commons Attribution 4.0 International license.

In the *F. prausnitzii-A. muciniphila* cocultures, *F. prausnitzii* 16S rRNA gene copy numbers decreased, and a small amount of butyrate appeared after 8 days of incubation. FISH staining revealed the presence of *F*. *prausnitzii* cells within the cocultures but confirmed its slow growth. Finally, within the *E*. *hallii*-*A. muciniphila* cocultures, low levels of butyrate started to build up after 8 days. This was associated with an increase in 16S rRNA gene copy numbers of *E. hallii* on day 8. Q-PCR and FISH staining showed an *A*. *muciniphila*-to-*E*. *hallii* ratio of 100:1 after 8 to 24 days (Fig. 3 and Table S1).

### Vitamin B_12_-dependent syntrophy between *E. hallii* and *A. muciniphila.*

Analyses of the metabolites produced in cocultures showed that in the *A. muciniphila*-*E. hallii* coculture, the proportion of succinate to propionate had shifted compared to the proportion in monocultures of *A. muciniphila* (Fig. 3). This was not observed in the other cocultures. Notably, 1,2-propanediol, found as a result of fucose degradation by *A. muciniphila* in monocultures, was not detected in the coculture with *E. hallii*.

### Conversion of propionate to succinate involves vitamin B_12_-dependent methylmalonyl-CoA mutase.

**FIG 4:**
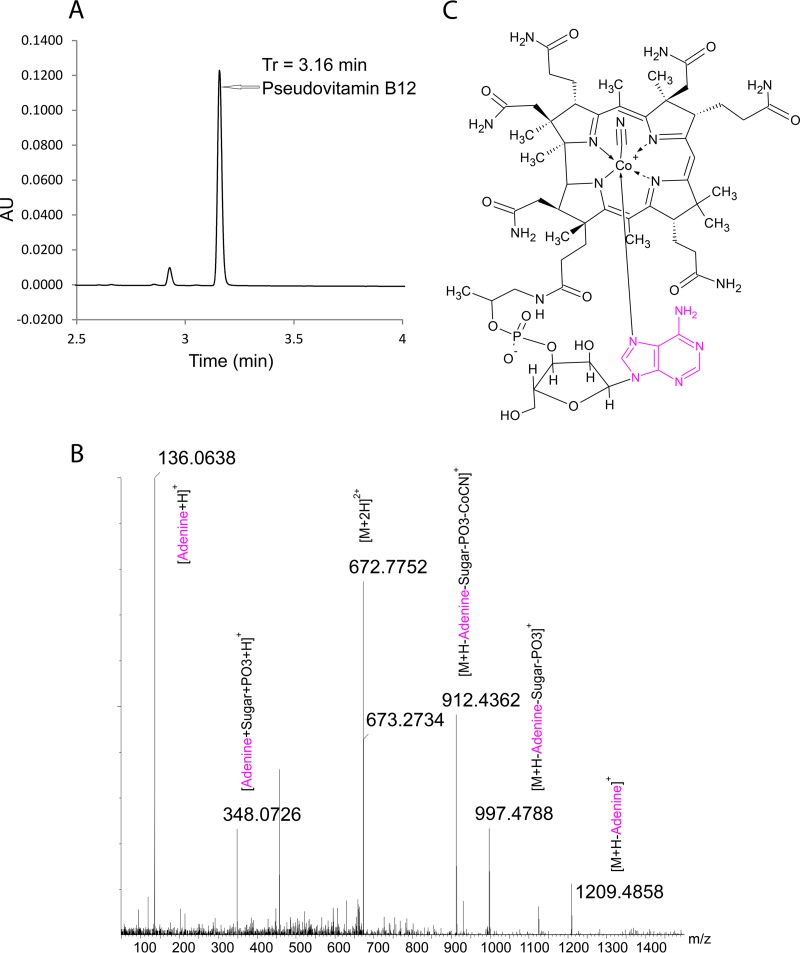
UHPLC-UV chromatogram of *E. hallii* vitamin B_12_. (A) Immunoaffinity-purified cell extract of *E. hallii* (in arbitrary units [AU]) is shown on the *y* axis, and time (in minutes) is shown on the *x* axis. Tr, retention time. (B) LC-MS/MS identified a peak at 3.16 min. (C) Chemical structure of pseudovitamin B_12_ from *E. hallii*.

Detailed mass spectroscopy analysis confirmed that *E. hallii* is capable of synthesizing a B_12_ vitamer in monocultures as described previously (24). Our analyses show that the structure of this vitamer (Fig. 4) is pseudovitamin B_12_, as the lower ligand contained adenine instead of 5,6-dimethyl benzimidazole (DMBI). No effect of DMBI addition was observed on the structure of the produced B_12_ vitamer.

To test the hypothesis that *A. muciniphila* can use the pseudovitamin B_12_ produced by *E. hallii* for the conversion of succinate to propionate, the effects of both purified *E. hallii* and commercially available vitamin B_12_ on *A. muciniphila* growth were tested. Indeed, the addition of pseudovitamin B_12_ and vitamin B_12_ resulted in significant lower succinate levels and significant higher propionate production. The addition of either vitamin B_12_ resulted in a profile identical to the profile observed for *A. muciniphila-E. hallii* coculture (Fig. 3).

**FIG 5:**
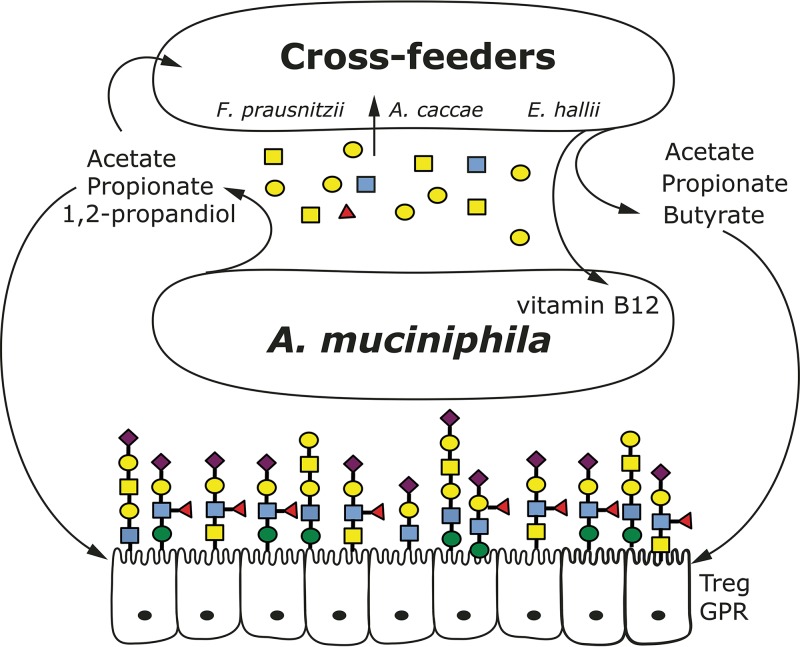
Schematic overview of mucus-dependent cross-feeding network. Keystone mucolytic bacteria, such as *A. muciniphila*, degrade mucin glycans resulting in oligosaccharides (mainly galactose, fucose, mannose, and GlucNac) and SCFAs (acetate, propionate, and 1,2-propanediol) that can be used for growth, as well as propionates, butyrate, and vitamin B_12_ production by cross-feeding partners. Treg GPR, regulatory T cell G-protein-coupled protein receptor.

These observations provide evidence for bidirectional metabolic cross-feeding between *A. muciniphila* and *E. hallii*. *A. muciniphila* liberates sugars from mucus and produces 1,2-propanediol for growth support of *E. hallii*. In return, *A. muciniphila* is provided with a vitamin B_12_ analogue used as a cofactor for the conversion of succinate to propionate via methylmalonyl-CoA synthase (Fig. 5). Apparently both vitamin B_12_ and pseudovitamin B_12_ can be used as a cofactor by *A. muciniphila* to activate the methylmalonyl-CoA synthase. Hence, the B_12_ vitamer produced by *E. hallii* is in the pseudovitamin B_12_ form and can be used by other intestinal microorganisms, but it has lower affinity than vitamin B_12_ for the human intrinsic factor (25).

## DISCUSSION

In spite of the great interest in metabolic conversions in the human gut, there is limited information on actual product sharing mechanisms and trophic dependencies of individual members of the intestinal microbiota. One such syntrophic relationship has been described for the species *Bacteroides thetaiotaomicron* and *Faecalibacterium prausnitzii* (26). *F. prausnitzii* can metabolize acetate produced by *B. thetaiotaomicron* to produce butyrate. This butyrate is then utilized by host epithelial cells and regulates host immunity via epithelial cell signaling, colonic T regulatory cells, and macrophages (19, 27). In addition, a few studies demonstrated the use of lactate and acetate produced by *Bifidobacterium* spp. by colonic butyrate producers (28–30). Specifically, this form of cross-feeding has been described for *Bifidobacterium adolescentis* and *F*. *prausnitzii* (30).

Moreover, cocultivation of amylolytic bacteria from the human colon, such as *Eubacterium rectale*, *B. thetaiotaomicron*, or *Bifidobacterium adolescentis*, with *Ruminococcus bromii* L2-63 can lead to increased starch utilization (31). In addition, coculturing of the non-starch-degrading species *Anaerostipes hadrus* with *R. bromii* has been shown to result in the removal of the reducing sugars that accumulate in *R. bromii* monocultures (32). Similarly, by stable isotope probing with ^13^C-labeled resistant starch has revealed a butyrogenic trophic chain between *R. bromii* and *E. rectale* in an *in vitro* human colon model (33, 34).

Various studies have coupled cooccurrence networks of bacteria to their genome content to model possible metabolic cross-feeding (22, 35). It should be noted that the studies discussed above all focus on cross-feeding that relies on diet-derived colonic sugars. However, mucin-derived sugars are the main source of energy for a group of microbiota members that can directly impact host cross talk at the mucosa (26). Mucus-dependent microbial networks at the mucosal layer would yield butyrate and other components with health benefits to the host (26). Our study supports the hypothesis that cross-feeding between microbiota members can take place when mucus is the only carbon source to support growth. Such mucosal trophic networks could determine host microbial cross talk in immune and metabolic regulation.

The mucosa-colonizing bacterium *A. muciniphila* is strongly correlated with a lean phenotype and increased barrier function (36–38). The correlation between *A. muciniphila* and host might depend on an additional microbial player. Indeed, we have shown that the mucus-degrading capacity of *A. muciniphila* may provide substrates to butyrate producers tested.

Two distinct types of trophic chains between *A. muciniphila* and butyrate-producing species were observed in this study. In the case of *A. caccae*, liberated sugars from mucus could sustain growth but *A. muciniphila*-derived acetate increased growth and metabolic production of butyrate even further, indicative of metabolic syntrophic interactions. In the case of *E. hallii*, a specific metabolic and cofactor syntrophic interaction was observed; pseudovitamin B_12_ affected the carbon flux within *A. muciniphila*, resulting in propionate production.

It is known from human studies that propionate delivered to the colon has various beneficial effects, including the regulation of satiety (39). Remarkably, *E. hallii* was able to utilize mucus sugars, in agreement with an earlier report (40). However, *E. hallii* had no clear advantage when acetate was present, possibly due to its own production of acetate when grown on mucus-derived sugars that already reached levels comparable to that of *A. muciniphila* monoculture.

Recently, it was reported that *E. hallii* is also able to use 1,2-propanediol for the production of propionate. Our data show the lack of 1,2-propanediol in the *A. muciniphila-E. hallii* coculture and supports the previous suggested syntrophic possibilities between intestinal microbes (24). 1,2-Propanediol is produced by *A. muciniphila* from fucose. As such, the presence or absence of fucose in the intestinal mucosa (FUT2 polymorphism) may help explain microbial networks at the mucosal layer (41). Furthermore, in coculture experiments with *A. muciniphila* and *F. prausnitzii*, low levels of butyrate were measured accompanied by the presence of cells and 16S rRNA copies of this butyrate producer as opposed to monocultures of the organism on the same medium (Table S2A). These results further indicate that the association of butyrate *Clostridium* cluster XIVa and IV species could indeed yield the production of butyrate as a result of a microbial metabolic network in the mucosal layer, which is poor in usable carbon sources.

10.1128/mBio.00770-17.2 TABLE S2  Growth and metabolic measurements of butyrogens. Download TABLE S2, PDF file, 0.1 MB. Copyright © 2017 Belzer et al. 2017 Belzer et al. This content is distributed under the terms of the Creative Commons Attribution 4.0 International license.

The fact that a changed metabolic profile for *A. muciniphila* in the presence of *E. hallii* was found is further evidence supporting a mutualistic syntrophic interaction. The availability of pseudovitamin B_12_ *in vivo* can be of importance for the microbial ecosystem as well as the host. Microorganisms are the only natural sources of the pseudovitamin B_12_ derivatives, and several intestinal microbes have been reported to contribute to the pseudovitamin B_12_ levels in the intestine (42). The approximate concentration of the cobalamin analogue adenine (as produced by *E*. *hallii*) is 164 ng/g (wet weight) of feces (43), and this is also in the range of what we found to be needed for *A. muciniphila* propionate induction (100 ng/ml). It is not clear whether pseudovitamin B_12_ can be used by intestinal cells. While the affinity of human intrinsic factor for pseudovitamin B_12_ is lower than that for vitamin B_12_, it is equally bound by transcobalamin and haptocorrin human corroid factors (25) and is not antagonistic to vitamin B_12_ (44), and it may be transported without intrinsic factor (45). Moreover, it has been shown that pseudovitamin B_12_ produced by *Lactobacillus reuteri*, also an abundant mouse intestinal bacterium, can alleviate vitamin B_12_ deficiency in mice (46, 47).

In summary, the present data indicate that pseudovitamin B_12_ is biologically active in *A. muciniphila* propionate metabolism that involves methylmalonyl-CoA mutase (48). Hence, the synthropic partners together produce a higher propionate-to-succinate ratio, and this in turn is beneficial for host cell metabolism. It also implies that stimulating or diminishing a keystone species, such as *A. muciniphila*, from the microbiota can have dramatic effect on a complete microbial network and associated host-microbe homeostasis. In this case, stimulating or administrating *A. muciniphila* within the intestine might benefit from addition of another organism or solely pseudovitamin B_12_ to stimulate the organism’s production of propionate and a healthy mucosal environment (Fig. 5).

Many gastrointestinal disorders have been associated with mucosal damage and lower gut barrier function. The fact that intestinal bacteria may have an impact on both these factors, either directly or via specific immune and metabolic stimulation, further emphasizes the importance of having the right bacteria at the right place. Loss of mucosal integrity and the associated mucobiome could be indicative of disease states and its development. *A. muciniphila* has been positively associated with a lean phenotype and beneficial metabolic gene regulation in human cell types (36, 49). Its presence might be essential for a mucosal adherent network of beneficial microorganisms that together prompt these effects of the host. As a matter of fact, weight loss studies usually report increased abundance of *Verrucomicrobia* (mainly *A. muciniphila*) as well as several other microbial species (50–52). Taken together, these results further indicate the possible importance of mucosa-associated microbial networks and their metabolic cross-feeding for regulation of host health-related parameters and prevention of disease.

## MATERIALS AND METHODS

### Bacterial growth conditions.

*Akkermansia muciniphila* MucT (ATTC BAA-835) was grown as described previously (17, 53). Purified mucin was prepared as follows. Ten grams of hog gastric mucin (type III; Sigma-Aldrich) was dissolved in 500 ml of 0.1 M NaCl (pH 7.8) containing 0.02 M phosphate buffer (0.02 M NaH_2_PO_4_ and Na_2_HPO_4_) (pH 7.8), stirring for 24 h at 4°C. After 1 h, the pH was adjusted to pH 7.2 using 1 M NaOH. After centrifugation, the supernatant was cooled on ice and precipitated with 60% (vol/vol) prechilled ethanol. After centrifugation, the pellet was dissolved in 0.1 M NaCl. These last two steps were repeated twice. After the last centrifugation step, the pellet was washed once with 100% ethanol, dissolved in 100 ml Milli-Q, and dialyzed using Spectra/Por 6 8,000-Da MWCO (molecular weight cutoff) protein dialysis with four changes. Last, the dialyzed mucin was freeze dried and dissolved in Milli-Q at a concentration of 5% (wt/vol). Mucin was added to the medium after autoclaving. The resulting purified mucin was tested for the absence of oligosaccharides. Incubations were performed in serum bottles sealed with butyl rubber stoppers at 37°C under anaerobic conditions provided by a gas phase of 182 kPa (1.5 atm) N_2_/CO_2_ (80/20 ratio). Growth was measured by a spectrophotometer as the optical density at 600 nm (OD_600_).

*Faecalibacterium prausnitzii* A2-165 was grown anaerobically at 37°C in YCFA medium supplemented with 33 mM acetate and 25 mM glucose (53). *Anaerostipes caccae* L1-92 (54) was grown anaerobically at 37°C in either PYG medium (DSMZ) or minimal medium (55) containing 25 mM glucose. *Eubacterium hallii* L2-7 was grown anaerobically at 37°C in YCFA medium without the addition of fatty acids (propionate, isovaleric acid, valeric acid, isobutyrate, and butyrate). Mucin sugar utilization was performed in minimal medium with or without the addition of 10 mM acetate. In some cases, the experiments were performed with mucin-derived single sugars (mannose [Sigma-Aldrich]), fucose (Sigma-Aldrich), galactose (Biochemika), *N*-acetylgalactosamine (Sigma-Aldrich), or *N*-acetylglucosamine (Sigma-Aldrich); these were used at a concentration of 25 mM. Growth was monitored for 24 h, and samples were collected regularly for OD_600_ and high-performance liquid chromatography (HPLC) analysis.

Coculture experiments were performed in minimal medium supplemented with mucus (17), and the medium was buffered to reduce pH changes due to fermentation products. Optimal coculture conditions were established as follows. *A. muciniphila* was added to media containing mucin, and the media containing bacteria were incubated for 8 h to reach measurable concentrations of acetate and liberate sugars. Subsequently, 10^8^ cells of *A. caccae*, *E. hallii*, or *F. prausnitzii* were added to the *A. muciniphila*-containing cultures. All cells had been washed twice with phosphate-buffered saline (PBS) before being added to the coculture to prevent carryover of products from the preculture. During the coculture, 0.15% mucin was added to the medium every 48 h to maintain sufficient substrate availability for *A. muciniphila*. All growth experiments were repeated a minimum of three times in duplicate.

### High-performance liquid chromatography.

For fermentation product analysis, 1 ml of bacterial culture was centrifuged, and the supernatant was stored at −20°C for high-performance liquid chromatography (HPLC) analysis. Substrate conversion and product formation were measured with a Thermo Scientific Spectrasystem high-performance liquid chromatography (HPLC) system equipped with a Varian Metacarb 67H column (300 by 6.5 mm) kept at 45°C and with 0.005 mM sulfuric acid as the eluent. The eluent had a flow rate of 0.8 ml/min, and metabolites were detected by determining the refractive index. Carbon balances were calculated by the amount of carbon of the products/amount of carbon of the substrate × 100%, using sugars and short-chain fatty acids (SCFAs) as measured by HPLC with biological triplicate samples and technical duplicate samples. We used theoretical CO_2_ calculations: 6 mol glucose yields 8 mol CO_2_, and 1 mol lactate yields 1 mol CO_2_.

### Ultrahigh performance liquid chromatography-mass spectrometry (UHPLC-MS).

For vitamin B_12_ analysis, *E. hallii* cells (0.2 g) were mixed with 10 ml of extraction buffer (8.3 mM sodium hydroxide and 20.7 mM acetic acid [pH 4. 5]) containing 100 µl of 1% NaCN. The vitamin was extracted in its cyano form by subjecting the mixture to a boiling water bath for 30 min. After cooling, the extract was recovered by centrifugation (6,900 × *g* for 10 min; Hermle, Wehingen, Germany) and finally purified by immunoaffinity column chromatography (Easy-Extract; R-Biopharma, Glasgow, Scotland). The reconstituted extract was analyzed for vitamin content using an HSS T3 C_18_ column (2. 1 by 100 mm; 1.8 µm) on a Waters Acquity UPLC (ultraperformance liquid chromatography) system (Milford, MA) equipped with a photodiode array detector (PDA) (210 to 600 nm) and interfaced to a high-resolution quadrupole time of flight mass spectrometer (QTOF; Synapt G2-Si, Waters). The eluent was a gradient flow (0.32 ml/min) of water (solvent A) and acetonitrile (solvent B), both acidified with 0.1% formic acid: 0 to 0.5 min (95 parts solvent A to 5 parts of solvent B [95:5]), 0.5 to 5 min (60:40), 5 to 6 min (60:40), and 6 to 10 min (95:5). The column was maintained at 30°C, and the UV detection was recorded at 361 nm. The MS analysis was done in positive ion mode with electrospray ionization, using a scanning range set for *m/z* of 50 to 1,500. The parent ions corresponding to the vitamin peak were further fragmented (tandem mass spectrometry [MS/MS]) and analyzed.

### Fluorescent *in situ* hybridization (FISH).

The following rRNA-targeted oligonucleotide probes were used: (i) Cy3-labeled universal EUB338 (5′-GCTGCCTCCCGTAGGAGT-3′), which is complementary to a conserved region of the bacterial 16S rRNA molecule specific to most eubacteria except phyla of *Plantomycetales* and *Verrucomicrobia* (17); and (ii) Cy5-labeled EUB338 III (5′-GCTGCCACCCGTAGGTGT-3′), the supplementary probes for eubacteria to target *Verrucomicrobia* (56).

### Cell fixation, *in situ* hybridization, DAPI staining, and microscopy.

Bacterial cultures (0.5 ml) were fixed overnight with 1.5 ml of 4% paraformaldehyde (PFA) at 4°C. Working stocks were prepared by harvesting bacterial cells by 5 min centrifugation at 8,000 × *g*, followed by resuspension in ice-cold phosphate-buffered saline (PBS) and 96% ethanol at a 1:1 (vol/vol) ratio. Three microliters of the PBS-ethanol working stocks were spotted into 18 wells (round wells with a 6-mm diameter) on gelatin-coated microscope slides. The slides were hybridized with the DNA probes by applying 10 μl of hybridization mixture per well, which contained 1 volume of probe mixture (probe concentration of 20 μM) and 9 volumes of hybridization buffer (20 mM Tris-HCl, 0.9 M NaCl, 0.1% SDS [pH 7.2]). The slides were hybridized for at least 3 h in a moist chamber at 50°C; this was followed by 30-min incubation in washing buffer (20 mM Tris-HCl, 0.9 M NaCl [pH 7. 2]) at 50°C for washing. The slides were rinsed briefly with Milli-Q and air dried. The slides were stained with a 4,6-diamine-2-phenylindole dihydrochloride (DAPI) mixture containing 200 μl PBS and 1 μl DAPI dye (100 ng/μl) for 5 min in the dark at room temperature, followed by Milli-Q rinsing and air drying. The slides were then covered with Citifluor AF1 and a coverslip. The bacteria on the slides were enumerated using an Olympus MT ARC/HG epifluorescence microscope. A total of 25 positions per well were automatically analyzed in three-color channels (DAPI, Cy3, and Cy5) using a quadruple band filter.

### Quantitative real-time PCR.

The abundances of *A. muciniphila* and butyrate producers in coculture were determined by quantitative real-time PCR. Bacterial cultures were harvested at 16,100 × *g* for 10 min. DNA extractions were performed using MasterPure Gram-positive DNA purification kit. The DNA concentrations were determined fluorometrically (Qubit dsDNA HS [double-stranded DNA high-sensitivity] assay; Invitrogen) and adjusted to 1 ng/μl prior to use as the template in quantitative PCR (Q-PCR). Primers targeting *A. muciniphila*, *A. caccae*, and *E. hallii* based on specific variable regions of the 16S rRNA gene (Table 3) were used for quantification. Standard template DNA was prepared from the 16S rRNA gene of each bacterium by amplification with primers 27F (F stands for forward) and 1492R (R stands for reverse). Standard curves were prepared with nine standard concentrations of 100 to 10^8^ gene copies μl^−1^. PCRs were performed in triplicate with iQ SYBR green supermix (Bio-Rad) in a total volume of 10 μl with primers at 500 nM in the wells on 384-well plates with the wells sealed with optical sealing tape. Amplification was performed with an iCycler (Bio-Rad) and the following protocol: one cycle of 95°C for 10 min; 35 cycles of 95°C for 15 s, 60°C for 20 s, and 72°C for 30 s each; one cycle of 95°C for 1 min; one cycle of 60°C for 1 min; and a stepwise increase of the temperature from 60 to 95°C (at 0.5°C per 5 s) to obtain melt curve data. Data were analyzed using the Bio-Rad CFX Manager 3.0.

**TABLE 3:** PCR primers used in this study and their amplification products

Bacterium	Primer	Primer sequence	Product size (bp)	Reference
*Akkermansia muciniphila*	AM1	5′ CAGCACGTGAAGGTGGGGAC 3′	327	57
	AM2	5′ CCTTGCGGTTGGCTTCAGAT 3′		

*Anaerostipes caccae* subgroup	OFF2555	5′ GCGTAGGTGGCATGGTAAGT 3′	83	58
	OFF2556	5′ CTGCACTCCAGCATGACAGT 3′		

*Eubacterium hallii* L2-7	EhalF	5′ GCGTAGGTGGCAGTGCAA 3′	278	59
	EhalR	5′ GCACCGRAGCCTATACGG 3′		

*Faecalibacterium prausnitzii*	FPR2F	5′ GGAGGAAGAAGGTCTTCGG 3′	248	59
	Fprau645R	5′ AATTCCGCCTACCTCTGCACT 3′		

### Statistics.

Statistics were performed using *t* test and corrected for multiple testing using false-discovery rate (FDR) correction for multiple comparisons. *P* values of <0.05 were considered significant.
