# Supplementary material for: Microbial Metabolic Networks at the Mucus Layer Lead to Diet-Independent Butyrate and Vitamin B12 Production by Intestinal Symbionts
Source: mBio. 2017 Sep 19;8(5):e00770-17. doi: 10.1128/mBio.00770-17 (PMC5605934; doi:10.1128/mBio.00770-17)
Supplement: TABLE S1 [file mbo004173482st1.pdf]

Co-culture	Time (day)	Cy5-labelled Mucin degrader		Cy3-labelled Butyrate producer		Ratio of Mucin degrader / Butyrate producer
		Avg	Stdev	Avg	Stdev	
A.muciniphila + A.caccae						
t=0h inoculation	1	8,73E+07	2,32E+07	4,90E+06	5,92E+06	17,82
	2	8,52E+07	1,37E+07	3,96E+06	1,08E+06	21,49
	4	3,25E+07	3,95E+06	2,33E+07	1,49E+07	1,40
	6	2,40E+07	2,54E+06	1,99E+07	1,97E+07	1,20
t=8h inoculation	1	1,31E+08	7,20E+06	2,59E+06	1,95E+06	50,57
	2	7,38E+07	2,73E+07	1,20E+07	1,27E+07	6,13
	4	3,04E+07	7,10E+05	1,04E+07	1,09E+07	2,92
	6	1,59E+07	1,11E+06	1,72E+07	1,63E+07	0,92
t=0h with washed inoculum	1	2,52E+08	1,25E+07	6,80E+05	7,46E+05	370,37
	2	2,20E+08	1,48E+08	2,25E+07	2,54E+07	9,79
	4	5,62E+07	3,34E+07	1,38E+07	8,90E+05	4,07
	6	2,68E+07	7,01E+06	3,76E+06	1,60E+06	7,13
t=8h with washed inoculum	1	2,24E+08	2,68E+07	5,46E+06	6,93E+06	40,98
	2	1,92E+08	4,81E+07	6,33E+06	6,26E+06	30,26
	4	3,55E+07	1,83E+07	3,77E+07	4,12E+07	0,94
	6	2,28E+07	-	2,38E+06	-	9,56
A.muciniphila + E.hallii	1	1,79E+07	1,06E+07	8,83E+06	4,31E+06	2,03
	2	5,08E+07	2,22E+07	3,61E+06	2,32E+06	14,10
	4	4,22E+07	2,50E+07	6,46E+06	3,06E+06	6,53
	6	1,91E+07	4,03E+06	5,11E+06	7,79E+05	3,74
A.muciniphila + F.prausnitzii	1	1,69E+07	1,03E+07	1,60E+07	8,56E+06	1,06
	2	7,48E+07	3,24E+07	8,04E+06	4,22E+06	9,31
	4	2,59E+07	5,63E+06	3,14E+06	8,81E+05	8,26
	6	2,34E+07	1,18E+07	6,84E+06	2,40E+06	3,42
