# Supplementary material for: Microbial Metabolic Networks at the Mucus Layer Lead to Diet-Independent Butyrate and Vitamin B12 Production by Intestinal Symbionts
Source: mBio. 2017 Sep 19;8(5):e00770-17. doi: 10.1128/mBio.00770-17 (PMC5605934; doi:10.1128/mBio.00770-17)
Supplement: TABLE S2 [file mbo004173482st2.pdf]

A

	OD600 Max		GlucNac		GalN		Galactose		Mannose		L-Fucose		1.2 propanediol		Acetate		Galactose		Glucose		Propionate		Succinate	
**growth on mucin media**	Average	Stdev	Average	Stdev	Average	Stdev	Average	Stdev	Average	Stdev	Average	Stdev	Average	Stdev	Average	Stdev	Average	Stdev	Average	Stdev	Average	Stdev	Average	Stdev
Unicoculated mucin media	-	-	-	-	-	-	-	-	-	-	-		-	-	-	-	-	-	-	-	-	-	-	-
A. muciniphila	1,24	0,23	-	-	0,03	0,06	0,12	0,12	-	-	0,03	0,05	1,63	0,39	16,10	3,53	-	-	-	-	0,47	0,69	13,79	2,72
A. caccae	0,10	0,19	-	-	-	-	-	-	-	-	-		-	-	-	-	-	-	-	-	-	-	-	-
E. hallii	0,015	0,08	-	-	-	-	-	-	-	-	-		-	-	-	-	-	-	-	-	-	-	-	-
F. prausnitzii	-0,055	0,02	-	-	-	-	-	-	-	-	-		-	-	-	-	-	-	-	-	-	-	-	-

N.D. = Not detected

B

	OD600 Max		GalNac		Mannose		Acetate		Butyrate		Formate		Glucose		Lactate		Propionate	
F. prausnitzii	Average	Stdev	Average	Stdev	Average	Stdev	Average	Stdev	Average	Stdev	Average	Stdev	Average	Stdev	Average	Stdev	Average	Stdev
YCFA + glucose	2,17	0,12	N.D.		N.D.		2,013092	4,09044	12,165432	2,6320785	20,57033	4,673598	-14,2957	5,439799	0,780973	0,423085	1,48768	1,264443
YCFA + mannose	0,07	0,01	N.D.		-3,77	2,31	-3,11	1,90	0,52	0,48	N.D.		N.D.		N.D.		-0,75	0,84
YCFA + GalNac	0,03	0,01	-1,90	0,96	N.D.		-3,61	4,02	0,48	0,37	N.D.		N.D.		N.D.		-1,33	0,80

C

	OD600 at 24h		L-Fucose		Acetate		Butyrate		Glucose		Isobutyrate		Lactate		Propionate		Succinate	
A. caccae	Average	Stdev	Average	Stdev	Average	Stdev	Average	Stdev	Average	Stdev	Average	Stdev	Average	Stdev	Average	Stdev	Average	Stdev
CP + Glucose	1,40	0,18	-	-	0,17	0,41	6,98	1,32	-	-	-	-	19,93	4,35	0,46	0,71	-	-
CP + Acetate	0,03	0,03	-	-	-	-	8,61	1,21	-	-	0,08	0,11	-	-	-	-	0,27	0,40
CP + Acetate & Lactate	N.D.	N.D.	N.D.	N.D.	N.D.	N.D.	N.D.	N.D.	N.D.	N.D.	N.D.	N.D.	N.D.	N.D.	N.D.	N.D.	N.D.	N.D.
CP + L-Fucose	0,01	0,02	22,38	1,04	-	-	0,82	0,13	-	-	-	-	0,06	0,16	0,93	1,45	-	-
CP + Acetate & L-Fucose	0,06	0,01	24,67	0,91	9,13	1,36	0,90	0,38	0,05	0,11	0,31	0,75	0,35	0,86	0,43	0,84	-	-
